# Auxin and Its Interaction With Ethylene Control Adventitious Root Formation and Development in Apple Rootstock

**DOI:** 10.3389/fpls.2020.574881

**Published:** 2020-10-15

**Authors:** Tuanhui Bai, Zhidan Dong, Xianbo Zheng, Shangwei Song, Jian Jiao, Miaomiao Wang, Chunhui Song

**Affiliations:** College of Horticulture, Henan Agricultural University, Zhengzhou, China

**Keywords:** auxin, indole-3-butyric acid, ethylene, adventitious root, apple rootstock 3/21

## Abstract

Adventitious root (AR) formation is indispensable for vegetative asexual propagation. Indole-3-butyric acid (IBA) functioned indirectly as precursor of IAA in regulating AR formation. Ethylene affects auxin synthesis, transport, and/or signaling processes. However, the interactions between auxin and ethylene that control AR formation in apple have not been elucidated. In this study, we investigated the effects of IBA and its interaction with ethylene on AR development in apple. The results revealed that IBA stimulated the formation of root primordia, increased the number of ARs, and upregulated expression of genes (*MdWOX11*, *MdLBD16*, and *MdLBD29*) involved in AR formation. Comparison of different periods of IBA application indicated that IBA was necessary for root primordium formation, while long time IBA treatment obviously inhibited root elongation. RNA-seq analysis revealed that many plant hormone metabolism and signal transduction related genes were differentially expressed. IBA stimulated the production of ethylene during AR formation. Auxin inhibiting ARs elongation depended on ethylene. Together, our results suggest that the inhibitory role of auxin on AR elongation in apples is partially mediated by stimulated ethylene production.

## Introduction

Apple (*Malus domestica* Borkh.) is one of the most economically important fruits worldwide and China has the largest area of apple tree cultivation (Wang et al., 2018). Apple trees are a combination of two genetically different parts: rootstock and scion. Rootstocks play a vital role in regulating the environmental adaptability and controlling the growth and development of apple trees (Atkinson et al., 2003; Gan et al., 2018). M9-T337, an excellent rootstock widely used in commercial orchards, could lead to dwarf tree architecture, early fruiting, and high fruit yield and quality (Zhang et al., 2016). Since the apple genome is highly heterozygous, vegetative propagation is widely used for experimental and commercial propagation in apple rootstock (Teixeira et al., 2019). The induction of adventitious roots (ARs) is a prerequisite for vegetative propagation. Therefore, identification and characterization of the factors regulating AR formation and development are essential for understanding and potentially manipulating AR formation.

The formation of ARs from non-root organs including stems or petioles is a complex developmental process (Li et al., 2009; Lei et al., 2018). AR formation from stem cuttings can be divided into four phases based on anatomical changes: dedifferentiation, induction, initiation, and ending with the emergence of roots (Atkinson et al., 2014; Legue et al., 2014; Mao et al., 2019; Druege et al., 2019). It is generally believed that the formation and development of ARs are affected by plant hormones, which provide a signaling network within the plant that determines cell fate and cell specialization and control a wide range of growth and developmental processes (Fattorini et al., 2017b; Mao et al., 2019).

Auxin has emerged as a central player in stimulating AR formation and is essential for the AR development (Della et al., 2013; Pacurar et al., 2014). Indole-3-butyric acid (IBA) is considered to be more stable and efficient than IAA in inducing AR formation in *in vitro* cultured explants and is widely used in clonal propagation (Bellini et al., 2014). Exogenous auxin and its analogs significantly improve AR formation of both greenwood and hardwood cuttings (Ludwig-Müller et al., 2005; Rahman et al., 2007; Xu et al., 2017; Wei et al., 2019). In *Arabidopsis*, IBA is a precursor of IAA. Conversion of IBA to IAA may generate NO and there is the hypothesis that it may act also independent on IAA (Veloccia et al., 2016; Fattorini et al., 2017b). Some of the auxin metabolism and signal transduction genes were reported to affect AR development. In *Arabidopsis thaliana*, auxin metabolism genes regulated AR formation through affecting the auxin content (Chen et al., 2016). Inhibiting the auxin polar transport significantly reduced the AR formation (Liu et al., 2018). Inhibiting polar auxin transport inhibited the early local rise of auxin, AR induction, and sink establishment in the root regenerating zone of petunia cuttings (Ahkami et al., 2013). The auxin response factor (ARF) AtARF6 and AtARF8 played the major roles that mediate auxin signaling during AR formation, while AtARF17 has a negative effect (Gutierrez et al., 2009). Auxin also induced the expression of *WUSCHEL-RELATED HOMEOBOX11/12* (WOX11/12) to promote expression of *AtLBD16* and *AtLBD29* to stimulate root primordia initiation and organogenesis (Liu et al., 2014; Hu and Xu, 2016).

In addition to auxins, ethylene is also a regulator of AR formation in a variety of plants. Ethylene is synthesized from the amino acid methionine by the consecutive action of S-adenosyl-L-methionine synthase, 1-aminocyclopropane-1-carboxylic acid (ACC) synthase, and ACC oxidase, functions in response to wounding in most plants, and promotes AR formation in cut tissues (Yang and Hoffman, 1984; Stepanova et al., 2005; Yu et al., 2019). ACC, a precursor of ethylene, can increase plant sensitivity to respond to endogenous ethylene (Cameron et al., 1979). Ethylene was also shown to regulate AR formation, likely through altering the transport and/or synthesis of auxin (Veloccia et al., 2016). AR formation and development are correlated with the expression levels of auxin and ethylene pathway genes in many species (Stepanova et al., 2005; Fattorini et al., 2017a; Liu et al., 2018). Ethylene was shown to induce the formation and growth of ARs by negatively affecting IAA synthesis but by positively enhancing the conversion of the IBA into active free IAA (Veloccia et al., 2016).

The role of IBA and ethylene in AR formation has been examined in a variety of plants, but the results were contradictory, with both positive and negative effects reported even in the same species (Yin et al., 2011; Druege et al., 2014; Lakehal and Bellini, 2019). Ethylene acts in a concentration-dependent and phase-specific manner, being either promotive or inhibitory in the AR formation (De Klerk and Hanecakova, 2008; Druege et al., 2019). AR formation in apple plants regulated by the interactions between auxin and ethylene remains unclear. In this study, four experiments were conducted to study the effects of auxin and ethylene on apple AR formation and elongation.

## Materials and Methods

### Plant Materials and Treatments

The tissue cultured M9-T337 apple rootstocks grew in a controlled-environment chamber maintained at 25/18°C (day/night) with a 16-h photoperiod (photosynthetically active radiation = 160 μmol⋅m^–2^⋅s^–1^), and relative humidity of approximately 70–80%. Stem cuttings of M9-T337 apple rootstock were used for all experiments. The treatment and sampling of the four experiments were as follows, and the flow chart was shown in Supplementary Figure S1.

#### Experiment 1

To investigate the roles of IBA in primordium induction of AR, stem cuttings of M9-T337 were divided into two groups: Control, for which the stem cuttings were transferred to 1/2 MS medium without IBA; and IBA treatment (IBA), for which the stem cuttings were transferred to 1/2 MS medium with 0.6 mg⋅L^–1^ IBA. Each treatment contained three biological replicates with at least 60 stem cuttings in each replicate, resulting in a total of 180 stem cuttings per group. The 0.6 mg⋅L^–1^ IBA was the optimal concentration to promote M9-T337 rooting that we screened earlier. After 0, 1, 3, 5, 7, and 27 days of treatments, the basal portions about 0.5 cm length from 30 stem cuttings at each time point were taken and frozen immediately in liquid nitrogen and stored at −80°C until subsequent use for RNA extraction.

#### Experiment 2

To examine the effect of IBA treatment time on the better development and growth of AR, stem cuttings of M9-T337 were divided into six groups; one group was cultivated on 1/2 MS medium without IBA and served as the control. The others were randomly divided into five groups and transferred to 1/2 MS medium with 0.6 mg⋅L^–1^ IBA and cultured in 1, 3, 5, 7, or 27 days, and then transferred to1/2 MS medium without IBA; these five groups were designated as 1d IBA, 3d IBA, 5d IBA, 7d IBA, and 27d IBA, respectively. At 8, 10, 13, 20, and 27 days after treatment, morphological parameters such as rooting percentage, the number of ARs per planted cutting, and mean length per AR were measured.

#### Experiment 3

Stem cuttings were divided into two groups, one group was cultivated in 1/2 MS medium with 0.6 mg⋅L^–1^ IBA for the entire experiment. The other group was transferred to 1/2 MS medium with 0.6 mg⋅L^–1^ IBA and cultured for 7 days, and then transferred to 1/2 MS medium without IBA; this group served as the control. Each treatment contained three biological replicates with at least 60 stem cuttings in each replicate, resulting in a total of 180 stem cuttings per group. After 7, 8, 12, 17, 21, and 27 days’ treatment, the basal portions were used for collecting the morphological and anatomical parameters, and transcriptome and gene expression analysis.

#### Experiment 4

In order to investigate IBA and its interaction with ethylene on AR formation and development, IBA, ethylene precursor ACC, and ethylene inhibitor AgNO_3_ were employed. Stem cuttings after 7 days of culturing on 1/2 MS medium with 0.6 mg⋅L^–1^ IBA were randomly divided into four groups and transferred to different medium: 1/2 MS medium; 1/2 MS medium with 0.6 mg⋅L^–1^ IBA; 1/2 MS medium with 0.6 mg⋅L^–1^ IBA and 3 mg⋅L^–1^ ACC; 1/2 MS medium with 0.6 mg⋅L^–1^ IBA and 3 mg⋅L^–1^ AgNO_3_. The four groups were respectively designated as the Control, IBA, IBA + ACC, and IBA + AgNO_3_. Each treatment contained three biological replicates with at least 60 stem cuttings in each replicate, resulting in a total of 180 stem cuttings per group. After 21 days of treatment, ethylene production, morphological parameters such as rooting efficiency, and the length and number of ARs per cutting were measured.

### Measurement of Morphological and Anatomical Parameters

The date of the first visible root emergence was recorded for each treatment. For anatomical examination, 1 mm thick cross-sections of stem cuttings were collected for fixation, paraffin embedding, and sectioning, which were conducted based on previously published protocols (Naija et al., 2008; Sundaramurthi and Smidts, 2013).

### RNA Extraction, cDNA Library Construction, and Sequencing

Total RNA was extracted from the basal portion of the cutting including the elongated roots according to the CTAB method (Chang et al., 1993). Total RNA integrity was assayed using the 28S and 18S rRNA bands on a 2% agarose gel. The sample libraries were prepared according the RNA-seq library constructed flow path and sequenced on an Illumina HiSeq 4000 system by Annoroad Gene Technology (Beijing) Co., Ltd. (Beijing, China). The RNA-seq resulted in paired-end reads, and each read contained 150 nucleobases. The raw sequence data from the sequencing machine were used for analysis. After filtering the low quality reads and contaminant sequences, the clean reads were aligned to the *Malus domestica* genome (GDDH13 Version 1.1^1^) (Daccord et al., 2017) using HISAT2 (Kim et al., 2015; Pertea et al., 2016). Stringtie software was used to assemble the transcripts (Pertea et al., 2015, 2016). Gene expression was calculated using the fragments per kilobase of transcript per million (FPKM) fragments mapped reads method (Mortazavi et al., 2008). DESeq2 software was used to estimate differentially expressed genes (DEGs) (Love et al., 2014). The FDR < 0.1 and |log_2_ (fold change)| ≥ 1 of genes between two samples were identified as DEGs (Benjamini and Yekutieli, 2001). Gene ontology (GO) and Kyoto Encyclopedia of Genes and Genomes (KEGG) enrichment analysis were used for the functional annotation of DEGs. The RNA-seq data were available at NCBI SRA (PRJNA643140).

### qRT-PCR Validation and Analysis

Quantitative real-time polymerase chain reaction (qRT-PCR) was used to analyze the gene expression. Primers were designed to amplify products of 100–300 bp using Primer 3 software (Supplementary Table S1). qRT-PCR was performed on the Bio-Rad CFX Connect Real-Time PCR Detection System. cDNAs were diluted to 100 ng and run in three technical replicates, with 1 μL template in a reaction volume of 20 μL. PCR amplification conditions were as follows: 95°C for 5 min for initial denaturation, then 45 cycles of 94°C for 20 s, 60°C for 20 s, and 72°C for 10 s. Fluorescence was measured at the end of each cycle. A melting curve analysis was performed to determine whether a single product was amplified. The apple *Actin* gene was used as an internal standard in the analysis. The relative expression level of each gene was calculated according to the 2^–ΔΔC_T^ method (Livak and Schmittgen, 2001). Values for mean expression and standard error (SE) were calculated from the results of three independent replicates.

### Ethylene Measurement

The ethylene production was measured according the methods of Liguori et al. (2004). Ten stem cuttings were weighed and sealed in a bottle for 1 h. The air (1 mL) from the jar was analyzed using a gas chromatograph with flame ionization detector (GC-2010 Plus, Shimadzu, Japan) (Li et al., 2017).

### Statistical Analysis

The significance between the treatment and control was determined by one-way ANOVA and significant differences between means were determined at the 5% level with Duncan test using the SPSS Statistics 17.0 software (IBM China Company Ltd., Beijing, China). Data are presented as means and SEs using Microsoft Excel.

## Results

### IBA Promoted the Root Primordium Formation of AR

**FIGURE 1:**
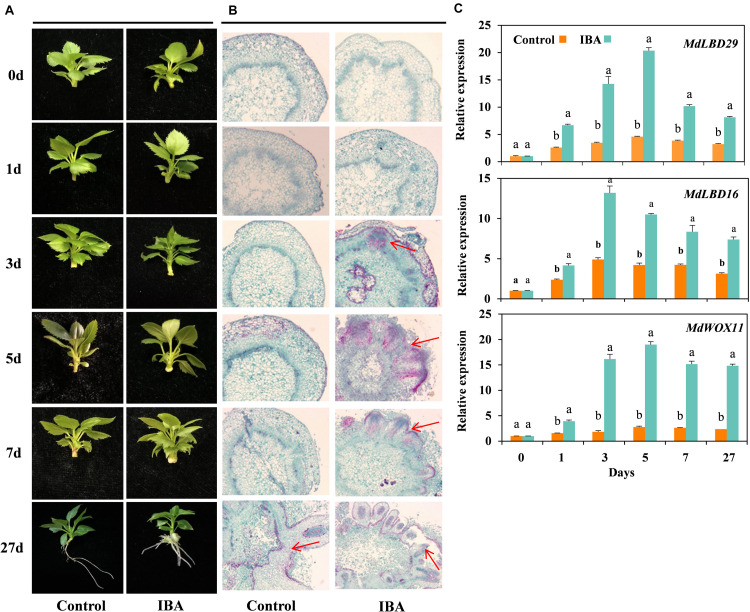
IBA stimulated the AR formation of M9-T337 cuttings. **(A)** Morphological and **(B)** anatomical observations of AR formation in stem cuttings of M9-T337 cultivated in 1/2 MS medium without (Control) or with (IBA) 0.6 mg⋅L^− 1^ IBA. **(C)** Expression levels of the genes involved in AR formation in the base of stem cuttings of M9-T337 cultivated in 1/2 MS medium without (Control) or with (IBA) 0.6 mg⋅L^− 1^ IBA. The arrows in **(B)** indicated the AR primordium. Values represent the mean ± SE of three biological replicates. Bars with the different letters indicate a significantly difference between treatments for a same date according to Duncan test (at *P* < 0.05).

Morphological changes of the tissue culture cuttings of M9-T337 in response to exogenous IBA treatment were continuously monitored during the rooting process and representative phenotypes were recorded (Experiment 1). As shown in Figure 1A, early signs of callus formation were observed at day 5 in IBA-treated cuttings. However, there were no signs of AR formation in control cuttings before day 7. Anatomical observations showed that IBA induced AR primordium formation at day 3; the AR primordium continued to divide and elongate, and then underwent tissue differentiation, and protruded outward through the cortex and epidermis to form AR at day 7 (Figure 1B). The stem tissue structures in the control group did not exhibit any significant changes. Based on the anatomical observations, M9-T337 AR formation induced by IBA was divided into four stages: stage 1 represents AR activation (first day), stage 2 represents AR induction (second to third day), stage 3 represents AR initiation phase (third to fifth day), and stage 4 represents ARs broken through the epidermis and emerged (fifth to seventh day).

WOX11, LATERAL ORGAN BOUNDARIES DOMAIN16 (LBD16), and LBD29 were reported to be involved in the first-step cell fate transition from procambium or parenchyma cell to a root founder cell (Liu et al., 2014). qRT-PCR showed that the expression levels of *MdWOX11*, *MdLBD16*, and *MdLBD29* significantly increased in the base of stem cutting treated with IBA compared to control (Figure 1C). It was suggested that IBA promoted the formation of AR by stimulating AR initial gene expression.

### IBA Inhibited AR Elongation

**FIGURE 2:**
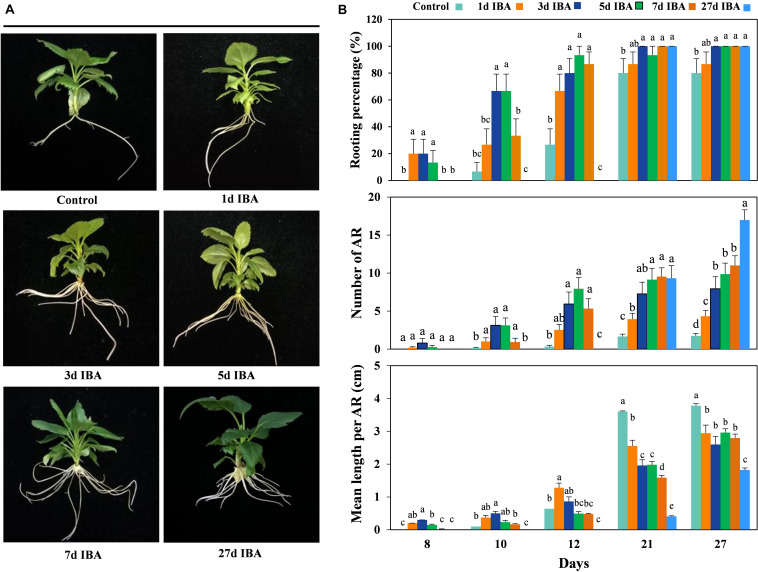
Effect of different periods of IBA application on AR development and growth. Morphology **(A)** and growth **(B)** of AR in stem cuttings of M9-T337 in the control, 1d IBA, 3d IBA, 5d IBA, 7d IBA, and 27d IBA groups. The control group is stem cuttings cultivated in 1/2 MS medium without IBA. 1d IBA, 3d IBA, 5d IBA, 7d IBA, and 27d IBA groups are stem cuttings cultivated in 1/2 MS medium with 0.6 mg⋅L^− 1^ IBA for 1, 3, 5, 7, or 27 days, and then transferred to1/2 MS medium without IBA. The photos in **(A)** were taken at the 27th day after treatment. Bars with the different letters indicate a significantly difference among treatments for a same stage according to Duncan test (at *P* < 0.05).

In order to verify the role of IBA in different stages of AR development, the stem cuttings grown in IBA rooting medium with different AR developmental stage were transferred to the 1/2 MS medium without IBA to observe AR growth and development (Experiment 2). The phenotypes and data of the apple rootstock cuttings responding to the IBA treatment during AR formation are presented in Figure 2. As the time of the stem cuttings stayed in the medium containing IBA increased, the rooting percentage and mean length per AR increased. With the exception of the control group and the 1d group, the rooting percentage of the other five groups reached 100%. This means that IBA was necessary in the AR induction stage.

**FIGURE 3:**
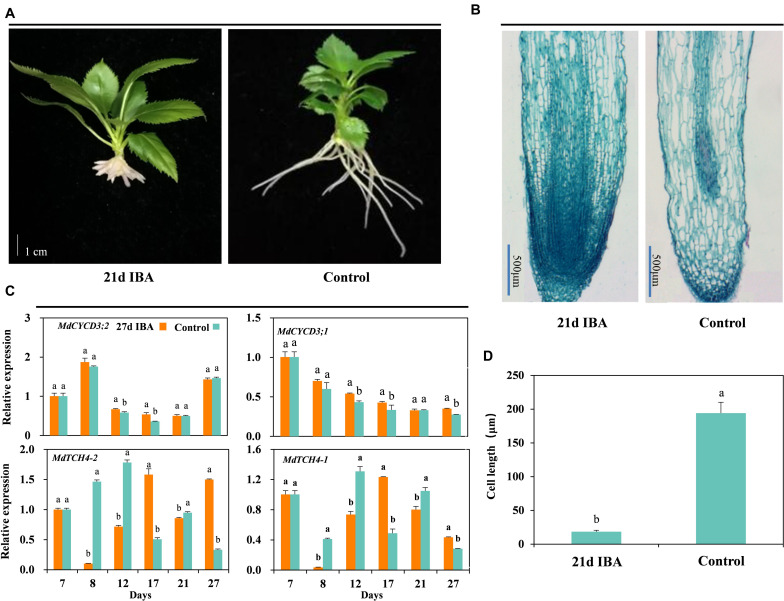
Effect of IBA on the AR cell elongation. Morphological **(A)** and anatomical structure **(B)** observations, **(C)** expression levels of the genes involved in cell cycle and elongation in the basal portion of the cutting including the elongated roots, and cell length **(D)** during AR growth and development in stem cuttings of M9-T337 of the control and 27d IBA groups. The control group is stem cuttings cultivated in 1/2 MS for the entire experiment. 27d IBA group is stem cuttings cultivated in 1/2 MS medium with 0.6 mg⋅L^− 1^ IBA for 27 days. The photos in **(A)** were taken at the 21st day after treatment. Bars with the different letters indicate a significantly difference between treatments for a same stage according to Duncan test (at *P* < 0.05). Scale bars in **(A)**, 1 cm. Scale bars in **(B)**, 500 μm.

The AR length of the IBA treated group was significantly shorter than the control (Figures 2B, 3A). We further analyzed anatomical structure of AR between the control and 27d IBA group. Anatomical observations showed that the cell length of the AR for 27d IBA treatment was shorter than the control (Figures 3B,D). Hence, IBA stimulated AR induction and formation, but inhibited AR elongation.

The relative expression of genes involved in cell cycle and elongation was also determined between the 27d IBA-treated and the control cuttings (Figure 3C). Expression of the cell cycle gene *MdCYCD3;2* was significantly higher in the 27d IBA-treated cuttings than the control cuttings at days 12 and 17. Expression of the cell cycle gene *MdCYCD3;1* was significantly higher in the 27d IBA-treated cuttings than the control cuttings at days 12, 17, and 27. *TCH4* encodes xyloglucan endotransglycosylase, which modifies xyloglucan chains in cell walls to control hormone-induced cell expansion and elongation (Xu et al., 1995). Expression level of the cell elongation genes *MdTCH4-1* and *MdTCH4-2* was significantly lower at days 8, 12, and 21 in the 27d IBA-treated cuttings than the control cuttings. In short, IBA increased cell division gene expression, but inhibited the cell elongation genes expression.

### Transcriptome Expression Analysis of the Effect of IBA on Adventitious Roots Elongation

To investigate the global transcriptome response of AR elongation to IBA during AR elongation, 12 cDNA libraries (IBA_12d-1, IBA_12d-2, IBA_12d-3, IBA_21d-1, IBA_21d-2, IBA_21d-3, Control_12d-1, Control_12d-2, Control_12d-3, Control_21d-1, Control_21d-2, Control_21d-3) were constructed from the basal portion of the cutting including the elongated roots of the control and IBA group after treatment for 12 and 21 days (Experiment 3). These libraries were sequenced using an Illumina HiSeq 4000 system and resulted in 44–71 million raw paired-end reads. After filtering the raw reads, we obtained 42–69 million clean reads for each library (Table 1). The Q30 clean reads percentage of all samples was above 93%. The clean reads were mapped to the apple genome (GDDH13) by HISAT2 software. The total mapping rates of all samples were about 93%.

**TABLE 1:** Quality statistics of RNA-seq data.

**Sample**	**Total raw reads**	**Total clean reads**	**Clean reads Q30 (%)**	**Mapping ratio (%)**
IBA_12d-1	56,468,532	54,168,884	94.45	92.46
IBA _12d-2	44,696,252	42,593,270	93.14	92.56
IBA _12d-3	71,105,568	69,230,626	93.9	93.03
IBA _21d-1	59,567,342	64,893,122	93.97	93.14
IBA _21d-2	63,020,458	59,311,920	94.02	93.29
IBA _21d-3	48,675,768	52,505,836	94.28	93.36
Control _12d-1	67,227,976	57,603,012	93.55	92.77
Control _12d-2	61,374,730	60,699,866	94.36	92.29
Control _12d-3	54,844,438	46,682,662	94.48	92.78
Control _21d-1	55,825,174	54,511,214	94.22	93.35
Control _21d-2	47,678,682	46,631,206	93.93	93.03
Control _21d-3	55,980,344	54,383,436	94.11	93.37

### Analysis of Differentially Expressed Genes

**FIGURE 4:**
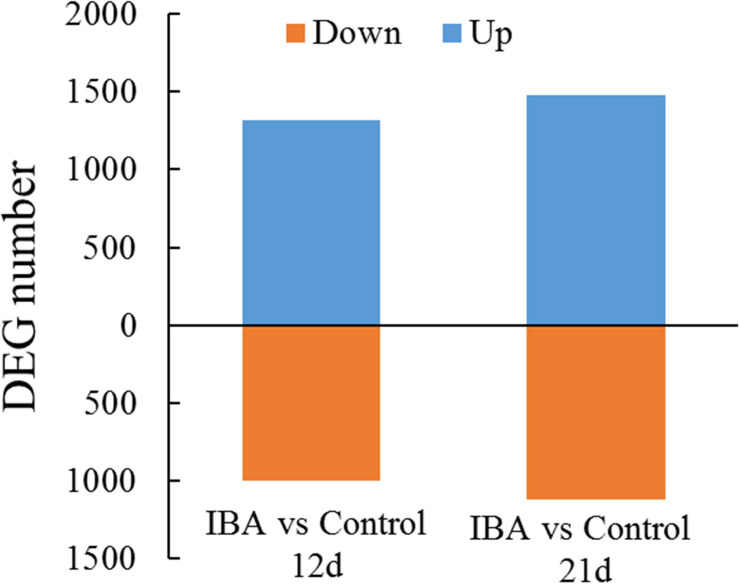
Comparison of differentially expressed genes between the stem base including elongating adventitious roots of control and IBA groups at days 12 and 21. The IBA group is stem cuttings cultivated in 1/2 MS with 0.6 mg⋅L^− 1^ IBA for the entire experiment. The control group is stem cuttings cultivated in 1/2 MS medium with 0.6 mg⋅L^− 1^ IBA for 7 days, and then transferred to1/2 MS medium without IBA. Blue: upregulated genes; orange: downregulated genes.

To identify DEGs between the control and IBA treatment, we investigated the gene expression levels using the FPKM mapped reads. The correlationship between the RNA-seq samples was shown in Supplementary Figure S2. There were 2,282 DEGs between IBA_12d and Control_12d, of which 966 were downregulated and 1,316 were upregulated (Figure 4). There were 2,592 DEGs between IBA_21d and Control_21d, 1,116 genes were downregulated and 1,476 genes were upregulated. Three fold change genes between IBA treatment and control were listed in Supplementary Tables S2, S3.

### Functional Annotation of DEGs

**FIGURE 5:**
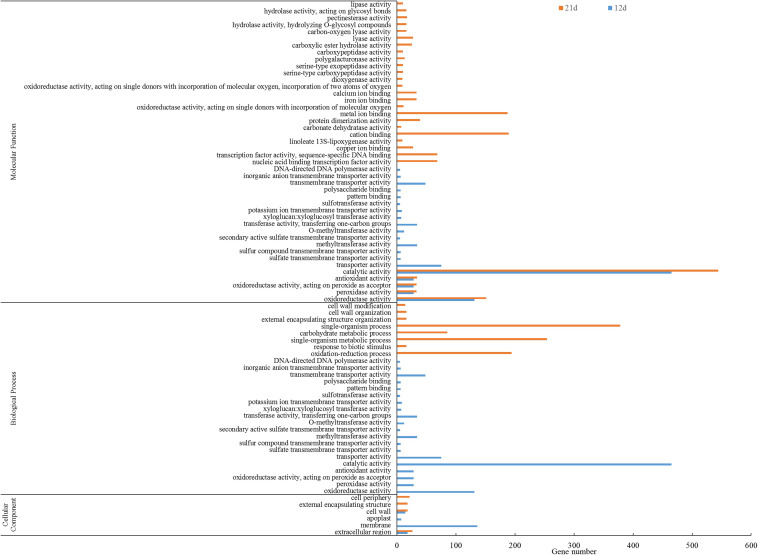
GO enrichment analysis of DEGs between the stem base including elongating adventitious roots of control and IBA groups at days 12 and 21. GO enrichment analysis of all the DEGs between the root of control and IBA. The IBA group is stem cuttings cultivated in 1/2 MS with 0.6 mg⋅L^− 1^ IBA for the entire experiment. The control group is stem cuttings cultivated 1/2 MS medium with 0.6 mg⋅L^− 1^ IBA for 7 days, and then transferred to1/2 MS medium without IBA.

Gene ontology annotation of the DEGs showed that 78 GO functional categories including molecular function (44), biological process (28), and cellular component were enriched (Figure 5). The five most significant biological processes were enriched in oxidation-reduction process (GO:0055114), single-organism metabolic process (GO:0044710), single-organism process (GO:0044699), response to biotic stimulus (GO:0009607), and carbohydrate metabolic process (GO:0005975). The most significant cellular component enrichments occurred in extracellular regions (GO: 0005576), cell wall (GO: 0005618), external encapsulating structure (GO: 0030312), extracellular region (GO: 0005576), and cell periphery (GO: 0071944). The molecular function enriched in oxidoreductase activity (GO: 0016491), peroxidase activity (GO: 0004601), oxidoreductase activity, acting on peroxide as acceptor (GO: 0016684), antioxidant activity (GO: 0016209), and nucleic acid binding transcription factor activity (GO: 0001071).

**FIGURE 6:**
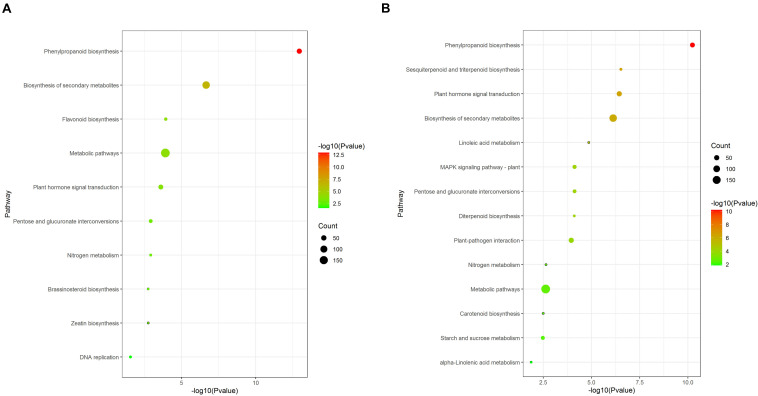
KEGG enrichment analysis of DEGs between the stem base including elongating adventitious roots of control and IBA groups at days 12 and 21. **(A)** KEGG enrichment analysis of DEGs between the control and IBA at 12 days. **(B)** KEGG enrichment analysis of DEGs between the control and IBA at 21 days. The IBA group is stem cuttings cultivated in 1/2 MS with 0.6 mg⋅L^− 1^ IBA for the entire experiment. The control group is stem cuttings cultivated in 1/2 MS medium with 0.6 mg⋅L^− 1^ IBA for 7 days, and then transferred to1/2 MS medium without IBA.

Kyoto Encyclopedia of Genes and Genomes pathway analysis results showed that some pathways were significantly enriched (Figure 6). Phenylpropanoid biosynthesis (ko00940), Biosynthesis of secondary metabolites (ko01110), Flavonoid biosynthesis (ko00941), Metabolic pathways (ko01100), and Plant hormone signal transduction (ko04075) were significantly enriched in the 12d samples (Figure 6A). Phenylpropanoid biosynthesis (ko00940), Sesquiterpenoid and triterpenoid biosynthesis (ko00909), Plant hormone signal transduction (ko04075), Biosynthesis of secondary metabolites (ko01110), and Linoleic acid metabolism (ko00591) were significantly enriched in the 21d samples (Figure 6B).

### DEGs Involved in Plant Hormone Metabolism and Signal Pathways

**FIGURE 7:**
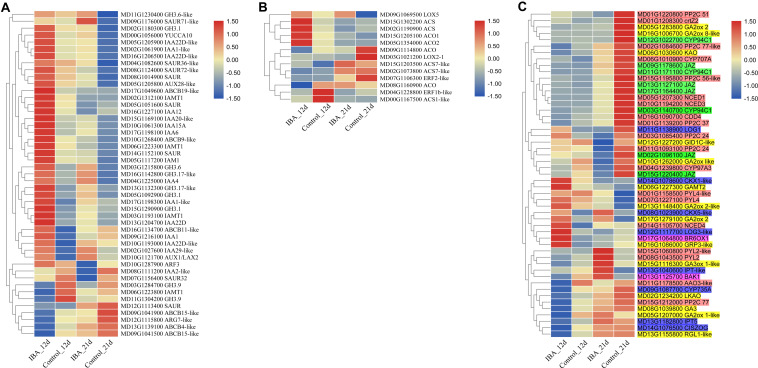
Heatmap of the plant hormone-related DEGs between the stem base including elongating adventitious roots of control and IBA groups at days 12 and 21. **(A)** Auxin. **(B)** Ethylene. **(C)** Other hormone. The IBA group is stem cuttings cultivated in 1/2 MS with 0.6 mg⋅L^− 1^ IBA for the entire experiment. The control group is stem cuttings cultivated 1/2 MS medium with 0.6 mg⋅L^− 1^ IBA for 7 days, and then transferred to1/2 MS medium without IBA. The FPKM value of the gene expressions from transcriptome is displayed in different colors. Red color means high expression and blue color means low expression.

Among the DEGs, plant hormone metabolism and signal transduction genes accounted for a large portion. There were 47 DEGs in the auxin metabolism, transport, and signal transduction pathway (Figure 7A). *IAA12*, *ABCB11-like*, *ABCB19-like*, *IAA1*, *IAA22D*, *GH3.1*, *AUX1/LAX2*, *IAA1-like*, *IAMT1*, *IAA22D-like*, *IAA15A*, *IAMT1*, *SAUR*, and *GH3.1* were upregulated in the 12d IBA treatment group. *SAUR36-like*, *GH3.6-like*, *SAUR71-like*, *IAA4*, and *YUCCA10* were upregulated in the 21d IBA treatment group. *IAMT1*, *GH3.9*, *ARG7-like*, *ABCB15-lik*e, *ABCB4-like*, and *ABCB15-like* were downregulated in the 12d IBA treatment group. *SAUR32*, *IAA2-like*, *SAUR*, and *ARF3* were downregulated in the 21d IBA treatment group. *AUX28-like*, *SAUR*, *SAUR72-like*, *GH3.6*, *IAA22D-like*, *ABCB9-like*, *IAA1-like*, *IAM1*, *GH3.17-like*, *GH3.1*, *IAA22D-like*, *GH3.17-like*, *IAA29-like*, *IAA6*, *IAA20-like*, and *IAMT1* were upregulated in the 12d and 21d IBA treatment groups.

For ethylene pathway, there were 13 DEGs in the ethylene synthesis and signal transduction pathways (Figure 7B). *ACS*, *ACS7-like*, *ACO1*, *ACS*, and *ACO2* were upregulated in the 12d IBA treatment group. *LOX5* was upregulated in the 21d IBA treatment group. *ACO*, *ACS7-like*, *ACS1-like*, and *ERF1b-like* were downregulated in the 12d IBA treatment group. *ACO*, *ERF2-like*, and *LOX2-1* were downregulated in the 21d IBA treatment group.

There were eight DEGs in the methyl jasmonate synthesis and signal transduction pathway including *JAZ* and *CYP94C1*. All these genes were downregulated in the 21d IBA treatment group (Figure 7C). There were 14 DEGs in the GA synthesis and signal transduction pathway. *GRP3-like* and *GAMT2* were upregulated in the 12d IBA treatment group. *GA2ox 2-like* and *GA3ox 1-like* were upregulated in the 21d IBA treatment group. *RGL1-like*, *GA2ox 1-like*, *GA3*, and *LKAO* were downregulated in the 12d IBA group. *GA2ox like*, *GID1C-like*, and *GA2ox 8-like* were downregulated in the 21d IBA treatment group. *GA2ox 2* and *KAO* was downregulated in the 12d and 21d IBA treatment groups. There were eight DEGs in the cytokinin synthesis and signal transduction pathway. *LOG3-like* and *CKX1-like* were upregulated in the 12d IBA treatment group. *CKX5-like* was upregulated in the 12d and 21d IBA treatment groups. *IPT-like* was upregulated in the 21d IBA treatment group. *IPT5* and *CISZOG* were downregulated in the 12d IBA treatment group. *CYP735A* was downregulated in the 12d and 21d IBA treatment group. *LOG1* was upregulated at the 12d, but downregulated at the 21d of the IBA treatment group. There were 19 DEGs in the ABA synthesis and signal transduction pathway. *NCED4* and *PP2C24* were upregulated in the 12d IBA treatment group. *PYL2*, *PYL4-like*, *PYL2-like*, and *PYL4* were upregulated in the 21d IBA treatment group. *PP2C 77* and *AAO3-like* were downregulated in the 12d IBA treatment group. *CDD4*, *CYP97A3*, *PP2C 37*, *NCED1*, *NCED3*, *PP2C 51*, *PP2C 24*, *CYP707A*, *crtZ2*, and *PP2C 56-like* were downregulated in 21d IBA treatment group. PP2C 77-like was downregulated in the 12d and 21d IBA treatment groups. There were two DEGs in the BR synthesis and signal transduction pathway. *BR6OX1* were upregulated in the 12d IBA treatment group. *BAK1* was upregulated in the 21d IBA treatment group.

### Ethylene Production During AR Development

**FIGURE 8:**
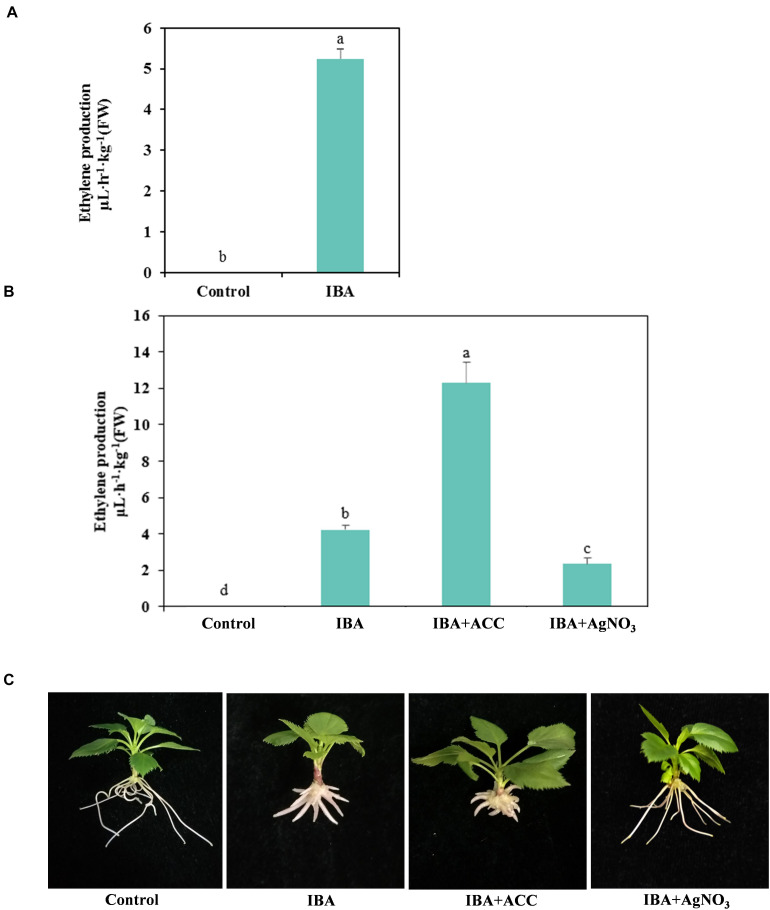
Relationship between IBA, ethylene production, and AR development. **(A)** Ethylene production in the IBA treatment group and the control group. The IBA group is stem cuttings cultivated in 1/2 MS with 0.6 mg⋅L^− 1^ IBA for the entire experiment. The control group is stem cuttings cultivated 1/2 MS medium with 0.6 mg⋅L^− 1^ IBA for 7 days, and then transferred to1/2 MS medium without IBA. **(B)** Ethylene production of ACC and AgNO_3_ treatment. **(C)** Morphological observation of ACC and AgNO_3_ treatment. The photos were taken at the 21st day after treatment. Ethylene production was measured at the 21 days after treatment. Control: 1/2 MS medium; IBA: 1/2 MS medium with 0.6 mg⋅L^− 1^ IBA; IBA + ACC: 1/2 MS medium with 0.6 mg⋅L^− 1^ IBA and 3 mg⋅L^− 1^ ACC; IBA + AgNO_3_: 1/2 MS medium with 0.6 mg⋅L^− 1^ IBA and 3 mg⋅L^− 1^ AgNO_3_. Bars with the different letters indicate a significantly difference among treatments by Duncan test (at *P* < 0.05).

Hormonal changes during M9-T337 stem cuttings AR formation induced by IBA have been studied (Lei et al., 2018). We further determined the gaseous hormone ethylene production (Experiment 3). The ethylene production of IBA treatment group was significantly higher than that growing in the 1/2 MS medium (Figure 8A). We further investigated the interactive effects of auxin and ethylene in regulating AR formation and development (Experiment 4). The ethylene inhibitor AgNO_3_ and ethylene precursor ACC were used as additives to be added into medium. ACC, the ethylene precursor, could be converted into ethylene in plants. As shown in Figure 8B, for the ACC group, more ethylene was produced. AgNO_3_ significantly reduced the ethylene production. AgNO_3_ promoted AR emergence and growth (Figure 8C and Table 2). While ACC slightly inhibited AR emergence and reduced AR number and length. Hence, ethylene inhibited AR elongation growth. Decreasing the ethylene perception in the IBA-treated cuttings, which further reduced ethylene production, increased AR length.

**TABLE 2:** Effect of ACC and AgNO_3_ on the AR emergence, AR number, AR length, and rooting efficiency in cuttings of M9-T337.

**Treatments**	**AR emergence (days)**	**AR number**	**AR length (cm)**	**Rooting efficiency (%)**
Control	9∼10	12.30 ± 1.33b	2.13 ± 0.09a	100.00 ± 0.00a
IBA	14∼15	17.40 ± 1.68a	0.84 ± 0.05b	100.00 ± 0.00a
IBA + ACC	16∼17	6.60 ± 0.66c	0.63 ± 0.03b	100.00 ± 0.00a
IBA + AgNO_3_	9∼10	13.80 ± 0.99b	2.10 ± 0.08a	100.00 ± 0.00a

*Root morphological parameters were measured at the 21 days after treatment. Data are mean values ± standard deviations; different letters in the same column indicate a significant difference among treatments according to Duncan test (at P < 0.05).*

### Expression of Auxin- and Ethylene-Related Genes

**FIGURE 9:**
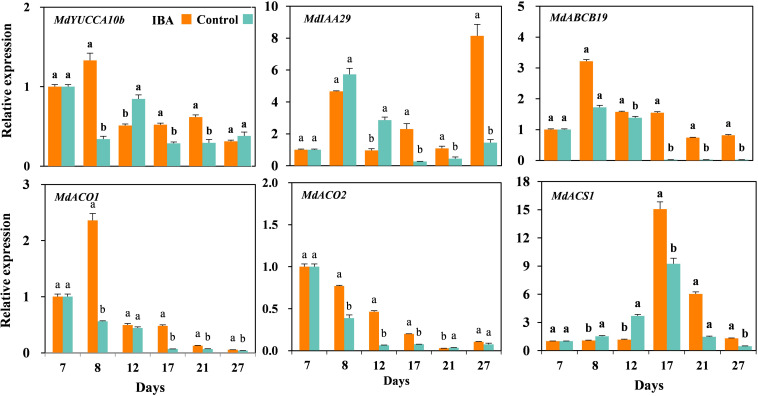
The expression levels of auxin- and ethylene-related genes during AR growth and development. The IBA group is stem cuttings cultivated in 1/2 MS with 0.6 mg⋅L^− 1^ IBA for the entire experiment. The control group is stem cuttings cultivated in 1/2 MS medium with 0.6 mg⋅L^− 1^ IBA for 7 days, and then transferred to1/2 MS medium without IBA. Values represent the mean ± SE of three biological replicates. Bars with the different letters indicate a significantly difference between treatments for a same stage according to Duncan test (at *P* < 0.05).

We further validated the auxin- and ethylene-related DEGs by qRT-PCR (Figure 9). The relative expression levels of auxin synthesis gene *MdYUCCA10* were significantly higher in the IBA treated plants than the control plants at days 8, 17, and 21, but significantly lower than control at the day 12. The expression of auxin signal transduction gene *MdIAA29* was significantly higher in the IBA treated plants than the control plants at days 17, 21, and 27, but significantly lower than control at days 8 and 12. The expression of auxin transport gene *MdABCB19* was significantly higher expression in the IBA treated plants than the control plants at days 8, 12, 17, 21, and 27. The relative expression levels of ethylene synthesis gene *MdACO1* were significantly higher in the IBA treated plants than the control plants at days 8, 17, 21, and 27. The relative expression levels of ethylene synthesis gene *MdACO2* were significantly higher in the IBA treated plants than the control plants at days 8, 12, and 17, but significantly lower than control at the day 21. The expression of ethylene synthesis gene *MdACS1* was significantly higher in the IBA treated plants than the control at days 17, 21, and 27, but lower than control plants at days 8 and 12.

## Discussion

Auxin is a plant hormone with versatile roles. The auxin analog IBA is used to stimulate rooting of cuttings in many plant species that are difficult to root (Karakurt et al., 2009; Aroonpong and Chang, 2015; Wei et al., 2019). Previous studies showed that exogenous IBA efficiently induced AR formation of both greenwood and hardwood cuttings (Karakurt et al., 2009; Gutierrez et al., 2012; Lei et al., 2018). In the present study, AR formation in the tissue cultured stem cuttings of M9-T337 apple rootstock was investigated. The results showed that exogenous IBA induced root primordium formation at day 3, the initial cells of root primordium continued to divide and differentiate, and subsequently protruded outward through the cortex and epidermis to form ARs at day 7. At the AR formation process, the AR induction key genes WOX11, LBD16, and LBD29 were induced significantly by IBA. There were no differences in rooting percentage between 3d, 5d, and 7d group. But transferring the 1 day IBA treated stem cutting to the IBA-free medium significantly reduced the rooting percentage. Hence, auxin was necessary in the AR induction stage. At the latter stage of AR formation, auxin was not necessary. In some other researches, it is found that after 96 h, auxin was no longer required in auxin induced stem cutting AR formation (De Klerk et al., 1999). In the M26, the third to seventh day after IBA treatment was a key period in AR formation process (Mao et al., 2019). Maybe different varieties have different responses of AR formation to IBA. In our study, stem cuttings growing in the 1/2 MS medium without IBA also could root, but the root number was significantly lower than the IBA treatment. ARs of stem cuttings growing in the 1/2 MS medium without IBA appeared later than IBA treatment. The endogenous IAA produced by the stem cutting itself promoted the formation of ARs. The promoting effect of exogenous IBA on AR formation can be expected to result from an enhanced endogenous auxin pool.

In the AR elongation stage, IBA inhibited AR elongation growth. The root anatomical structure showed that the cell length of the AR growing in the medium containing IBA was shorter than in the 1/2 MS. The expression of cell elongation genes was significantly lower in the IBA treated samples. But the cell cycle genes were higher in the IBA treated samples. Optimal auxin concentration for AR induction was inhibitory for root growth (De Klerk et al., 1999). Hence, IBA stimulated the root cell division, but decreased the root length by reducing the cell length. Based on these results, it is recommended that after the stem cuttings were treated in IBA induction rooting medium for 7 days, it would be better to transferred to medium without IBA to promote the growth of ARs.

There were 2,282 DEGs between IBA 12d and Control 12d and 2,592 DEGs between IBA 21d and Control 21d. As IBA is an auxin analog, auxin metabolism and signal transduction genes were significantly enriched. Some early auxin response genes were significantly differentially expressed between IBA treatment group and the control. Some of the GH3 genes that may act as acyl acid amido synthetases conjugating IBA or conjugate IAA with amino acids were upregulated. *GH3.9* mutant *A. thaliana* plants showed longer primary root length and increasing sensitivity to IAA-mediated root growth inhibition (Khan and Stone, 2007). The *ABCB* family genes were significantly differentially expressed between IBA treatment and the control. *ABCB1* and *ABCB19* function in IAA transport, and *ABCB19* overexpression *Arabidopsis* plants formed more AR than the wild type when the primary roots were excised (Sukumar et al., 2013). AUX/IAA proteins act as negative regulators by repressing *ARFs* (Tiwari et al., 2004). All *AUX/IAA* genes were upregulated in the IBA treated samples. *IAA14* and *IAA12* act in *Aux/IAA-ARF* modules *SLR/IAA14-ARF7-ARF19* and *BODENLOS/IAA12-ARF5*, controlling the LR initiation and patterning process (Vanneste et al., 2005). The high expression of *AUX/IAA* genes in the IBA treated sample may be a feedback regulation to regulate excessive auxin response. Also in petunia cuttings, higher endogenous auxin levels were associated with higher expression of Aux/IAA genes, which was explained by the auxin-induced degradation of the Aux/IAA proteins that leads to subsequent enhanced transcription of respective genes encoding the same Aux/IAA proteins (Yang et al., 2019). Based on these results, it is suggested that IBA is important for AR formation and IBA might be able to directly affect the expression of key auxin metabolism and signal transduction genes directly involved in AR formation.

Ethylene could affect AR development in cuttings of different plant species (Wei et al., 2013; Veloccia et al., 2016). Ethylene positively or negatively regulates AR formation, probably through the modulation of auxin synthesis/action/transport (Yin et al., 2011; Druege et al., 2014). AR formation in apple plants regulated by the interactions between auxin and ethylene remains unreported. In the present study, we found 13 DEGs involved in the ethylene synthesis and signal pathways. Ethylene synthesis genes *MdACO* and *MdACS* were upregulated in the IBA treated samples. IBA stimulated the ethylene production of stem cuttings during AR development. Inhibiting the ethylene production by the ethylene inhibitor AgNO_3_ could increase AR length. Auxin and ethylene had synergistic effects on plant development (Růžicka et al., 2007). Auxin induces ethylene biosynthesis by upregulation of ACC synthase (Wang et al., 2005). Ethylene influences auxin levels by regulating the expression of auxin synthesis genes *WEAK ETHYLENE INSENSITIVE* (*WEI2*, *WEI7*, and *WEI8*) (Stepanova et al., 2005). In the presence of 10 μM ACC, root elongation in the wild type *Arabidopsis* seedlings was almost completely inhibited. Ethylene promotes or inhibits rooting depending on the stage in the rooting process and reflects the stimulation effects during the initial period, but the inhibitory effects during later stages of AR development (De Klerk et al., 1999; De Klerk and Hanecakova, 2008). Ethylene inhibits root growth primarily by affecting cell elongation but not root meristem activity (Růžicka et al., 2007). In addition to ethylene, auxin also inhibits root elongation (Rahman et al., 2007; Bai et al., 2017). The cell length of IBA-treated AR was significantly shorter than the control. But in the presence of ethylene inhibitor AgNO_3_, the phenotype of IBA inhibiting root elongation was partially rescued. Therefore, high concentrations of auxin and ethylene inhibited the AR elongation. The inhibitory effects of high auxin supply on AR elongation are at least partially mediated by ethylene.

**FIGURE 10:**
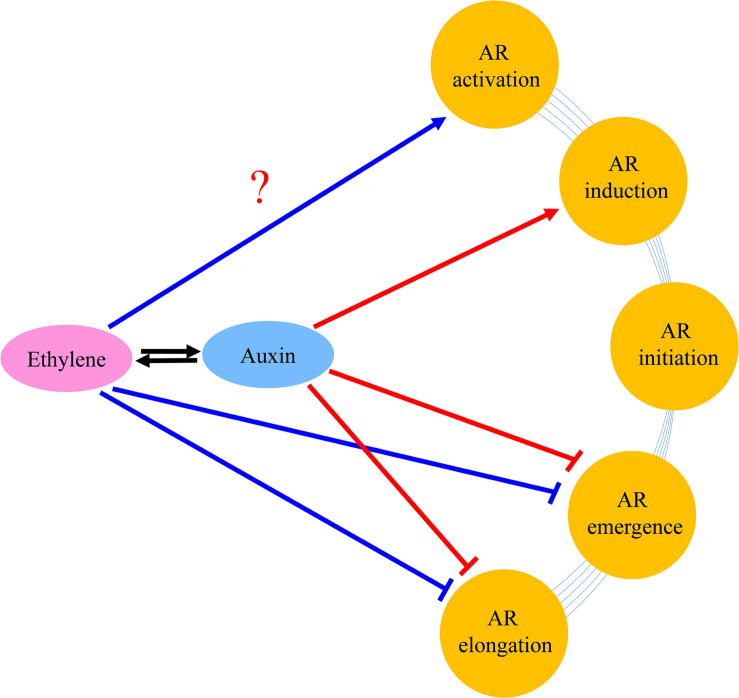
Tentative model of the regulation networks about auxin and interaction with ethylene during AR formation and development. Arrows and inhibition lines represent positive and negative interactions, respectively. The question mark indicates possible regulation mode.

Based upon the results of the current study and former researches, we present a model to explain the roles of auxin and ethylene in AR formation and development (Figure 10). Auxin stimulated AR formation in the AR induction stage, while a high concentration of auxin had a negative effect on the AR emergence and AR growth. Auxin inhibited AR elongation partially dependent on ethylene. Auxin stimulated ethylene production, and decreased AR length by inhibiting cell elongation genes.

## Data Availability Statement

The RNA-seq data are available at NCBI SRA (PRJNA643140).

## Author Contributions

TB and CS conceived and designed research. ZD conducted experiments. XZ, SS, JJ, and MW revised the manuscript and edited language. TB and CS analyzed the data and finalized the manuscript. All authors read and approved the manuscript.

## Conflict of Interest

The authors declare that the research was conducted in the absence of any commercial or financial relationships that could be construed as a potential conflict of interest.

## Footnotes

1 https://iris.angers.inra.fr/gddh13/the-apple-genome-downloads.html

## Supplementary Material

Supplementary Figure 1 Plant materials and experiment design.

Supplementary Figure 2 Correlationship between the RNA-seq samples.

Supplementary Table 1 Primer sequences for qRT-PCR.

Supplementary Table 2 Genes with three-fold change expression between IBA and Control at 12 days.

Supplementary Table 3 Genes with three-fold change expression between IBA and Control at 21 days.
