# Supplementary figures and images for: Auxin and Its Interaction With Ethylene Control Adventitious Root Formation and Development in Apple Rootstock
Source: Front Plant Sci. 2020 Oct 15;11:574881. doi: 10.3389/fpls.2020.574881 (PMC7593273; doi:10.3389/fpls.2020.574881)

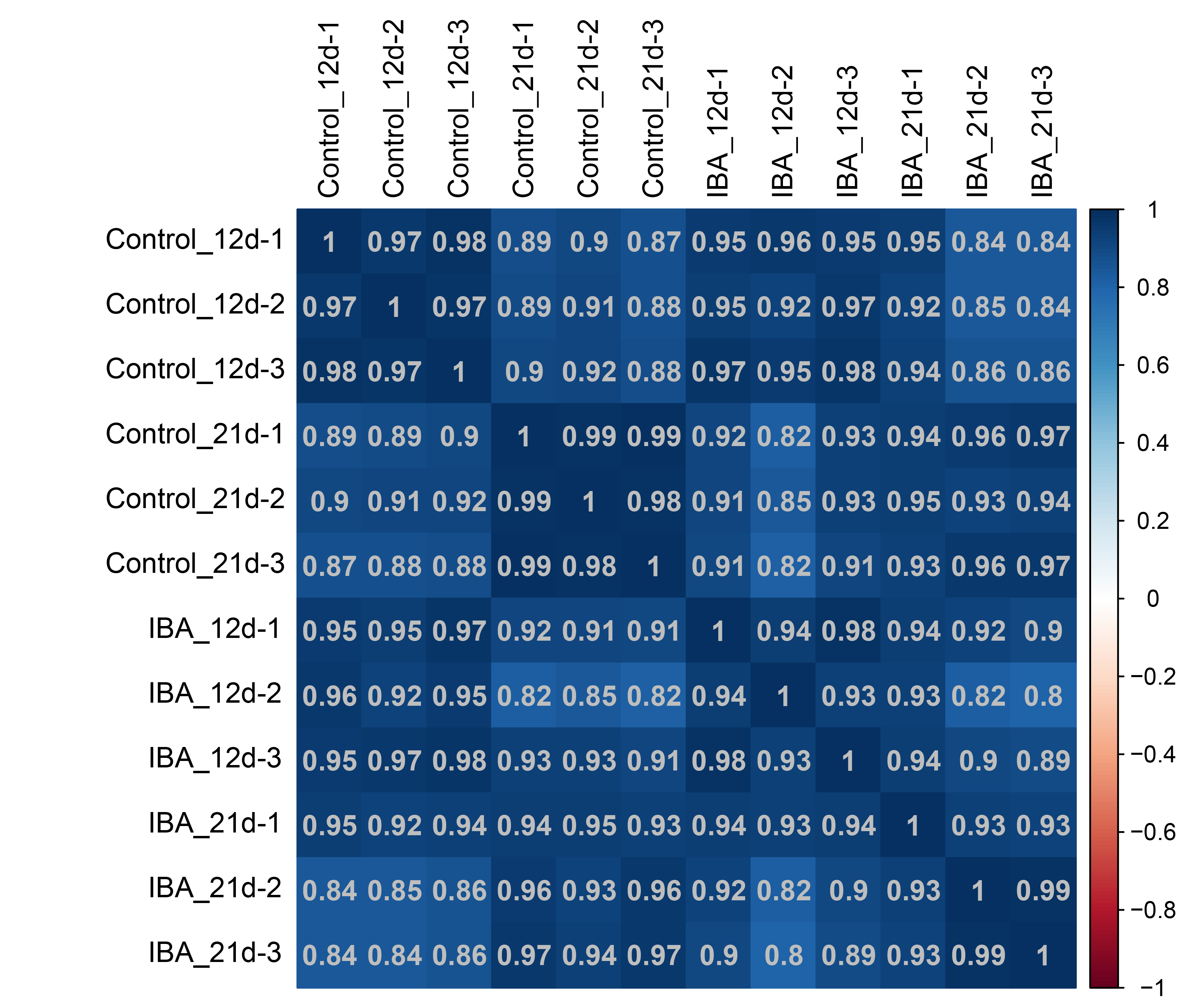

Supplement: Supplementary Figure 1 — Plant materials and experiment design. [file Image_1.JPEG]

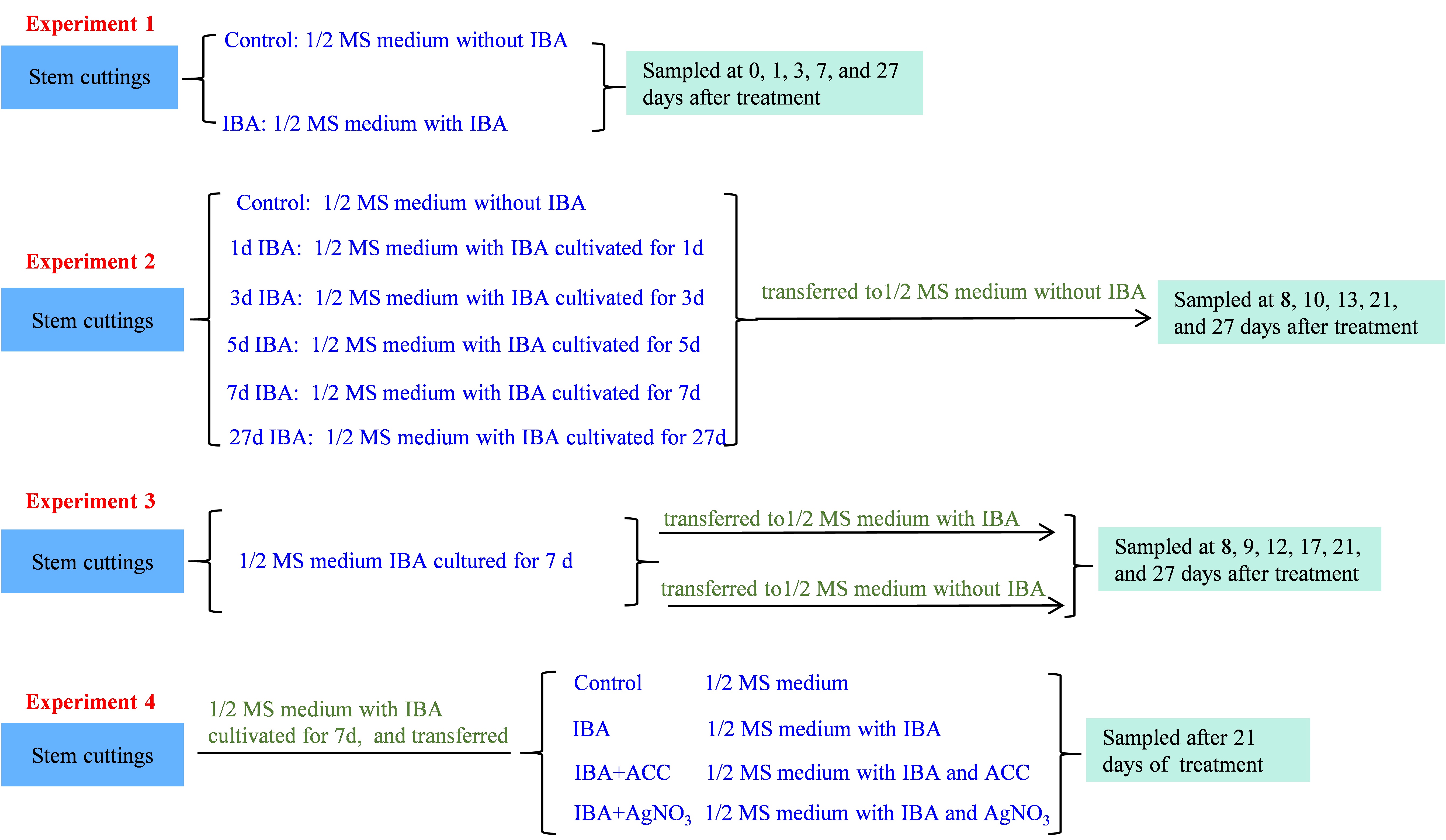

Supplement: Supplementary Figure 2 — Correlationship between the RNA-seq samples. [file Image_2.JPEG]
